# Current Challenges for Biological Treatment of Pharmaceutical-Based Contaminants with Oxidoreductase Enzymes: Immobilization Processes, Real Aqueous Matrices and Hybrid Techniques

**DOI:** 10.3390/biom12101489

**Published:** 2022-10-15

**Authors:** Helena Sá, Michele Michelin, Teresa Tavares, Bruna Silva

**Affiliations:** 1 CEB—Centre of Biological Engineering, University of Minho, 4710-057 Braga, Portugal; 2 LABBELS—Associate Laboratory, University of Minho, Campus Gualtar, 4710-057 Braga, Portugal

**Keywords:** oxidoreductases, pharmaceuticals, enzyme immobilization, biodegradation, wastewater, hybrid techniques

## Abstract

The worldwide access to pharmaceuticals and their continuous release into the environment have raised a serious global concern. Pharmaceuticals remain active even at low concentrations, therefore their occurrence in waterbodies may lead to successive deterioration of water quality with adverse impacts on the ecosystem and human health. To address this challenge, there is currently an evolving trend toward the search for effective methods to ensure efficient purification of both drinking water and wastewater. Biocatalytic transformation of pharmaceuticals using oxidoreductase enzymes, such as peroxidase and laccase, is a promising environmentally friendly solution for water treatment, where fungal species have been used as preferred producers due to their ligninolytic enzymatic systems. Enzyme-catalyzed degradation can transform micropollutants into more bioavailable or even innocuous products. Enzyme immobilization on a carrier generally increases its stability and catalytic performance, allowing its reuse, being a promising approach to ensure applicability to an industrial scale process. Moreover, coupling biocatalytic processes to other treatment technologies have been revealed to be an effective approach to achieve the complete removal of pharmaceuticals. This review updates the state-of-the-art of the application of oxidoreductases enzymes, namely laccase, to degrade pharmaceuticals from spiked water and real wastewater. Moreover, the advances concerning the techniques used for enzyme immobilization, the operation in bioreactors, the use of redox mediators, the application of hybrid techniques, as well as the discussion of transformation mechanisms and ending toxicity, are addressed.

## 1. Introduction

Water is one of the primary resources in an ecosystem and human sustenance. Intensive urbanization has led to increased water consumption for domestic, agricultural and industrial uses, resulting in the generation of tremendous amounts of wastewater [1]. Water pollution is considered a global problem as it involves rivers, lakes and oceans, and ultimately drinking water throughout the world [2]. Consequently, a lot of attention is being devoted to the lack of quality of water bodies due to the elevated levels of organic and inorganic chemicals and complex compounds in wastewater [3]. 

A particular concern about a multitude of newly identified compounds of anthropogenic or natural origin in the environment has gained significant attention due to their potentially serious threats to human health and ecosystems [3]. These compounds, termed emerging contaminants (EC), consist of substances that are currently not controlled in the environment, but have high toxicological potential for both ecosystems and human health, as well as can cause ecological damage [4,5]. Among these compounds there are pharmaceuticals and personal care products, pesticides, steroid hormones and industrial chemicals [6].

Hundreds of tons of pharmaceutical compounds are annually dispensed and consumed worldwide [7]. Pharmaceutical active compounds (PhAC) are a group of hazardous contaminants and their fate in water bodies is an increasing environmental concern due to their high consumption, their persistence and activity, and their potential adverse effects on environmental health [8].

Conventional wastewater treatment plants (WWTP) comprise conventional technologies used to remove a wide variety of pollutants. However, these processes are unable to completely remove organic micropollutants present in urban wastewater [9]. Research efforts have led to innovation in water treatment technologies using advanced oxidation process, photodegradation, electrochemical degradation, Fenton’s process, phase-changing technologies (e.g., adsorption using activated carbon and membrane technology) and biological processes (e.g., activated sludge and biological filtration) [4,10,11,12,13,14]. Although research has established favorable outcomes, these treatments have inherent limitations, such as the generation of a considerable amount of waste and loss of pollutants into the environment during membrane separation and adsorption on activated carbon, the treatment of the membrane concentrate, the formation of potentially more toxic by-products, low effectiveness, the requirement of complex operational and expensive plant requirements [4,15,16,17]. 

In the coming years it is essential to invest in innovative, efficient, environmentally friendly and less expensive methods for the abatement of pollutants from water. Enzymatic biotransformation of organic pollutants has progressively attracted interest in the development of alternative treatments [18,19]. The biocatalytic degradation of PhAC with enzymes may offer enormous potential for water remediation due to high catalytic activity, low energy input requirement and mild conditions of operation in the presence of variable concentrations of pollutants [20]. Furthermore, the specificity of the enzymatic methods over a wide range of pollutants leads to the minimization of undesirable products and the by-products are generally less or non-toxic [21]. 

Oxidoreductase enzymes are well known for the degradation of recalcitrant pollutants [22]. However, this biocatalytic approach has not been scaled up yet due to some barriers, namely rapid enzyme denaturation, lack of free enzyme reusability and requirement of large quantities of catalyst, which will enhance the overall cost and limit their application [23]. Recently, improvement in the catalytic stability and reusability of biocatalysts, and consequently a significant reduction in treatment cost, has been achieved by immobilization technology on several different supporting matrices [24]. 

Therefore, the immobilization of biocatalysts on sustainable supports is a promising approach to removing target PhAC in aqueous matrices, which could be used as a cost-effective and eco-friendly alternative to conventional treatments used in WWTP. In this context, this work aims to address the recent advances in the application of oxidoreductases, namely laccase (Lac), to remove pharmaceuticals from aqueous media. The current review provides a discussion about the biotransformation challenges concerning enzymes mediators and immobilization, bioreactors, hybrid techniques, as well as biocatalytic transformation mechanisms and ending toxicity. To the best of our knowledge, this work may serve as a base to close some of the existing literature gaps about enzymatic degradation processes of PhAC, aside from benefiting future developments. 

## 2. Occurrence and Toxicity of PhAC

PhAC are chemical substances used to diagnose, prevent, treat and change diseases, infections, or discomforts [25]. A wide variety of human and animal medicines such as analgesics, non-steroidal anti-inflammatory drugs (NSAID), antibiotics, lipid regulators, beta-blockers, synthetic hormones and X-rays contrast media has become essential to ensure the health and well-being of the population and, therefore, to increase their life expectance [26]. 

**Figure 1:**
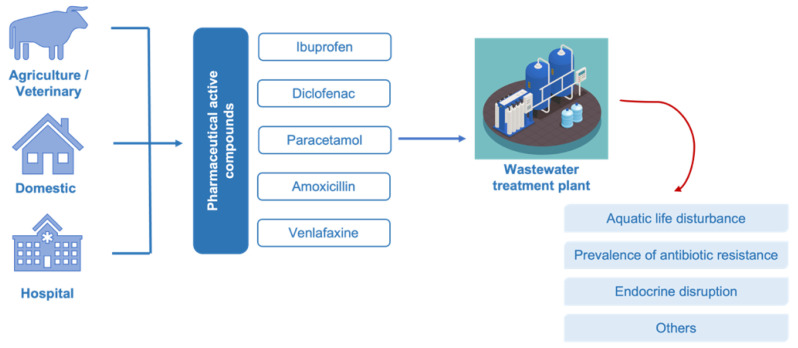
Path of pharmaceuticals active compounds from the use to the disposal in the environment with some of the ecological side effects.

The consumption of PhAC is set to increase in future years, due to population aging and improvements in health standards. Accordingly, several pharmaceuticals have been found in wastewater effluents, drinking water and in rivers at low concentrations ranging from ng/L to μg/L [3,27]. As can be seen in Figure 1, PhAC can be generated by different sources (e.g., agricultural activities, domestic and hospitals), as well as are designed to be biologically active and may affect non-target organisms. Although pharmaceuticals are usually designed with a single mechanism of action and target, non-target organisms may have receptors and, therefore, unexpected effects may result from unintended exposure [28]. 

The prevalence of antibiotics in aquatic environments has become an important concern worldwide with the increasing occurrences of antibiotic-resistant bacteria and resistance genes in wastewater and even drinking water, due to their intensive use for veterinary, agricultural and human medical proposes [29,30]. Another major concern is their ability to interfere with the endocrine system to provoke undesired effects/disruption of homeostasis [3,31]. Continuous exposure to estrogens that are able to mimic and/or antagonize the effects of endogenous hormones produces adverse effects on the reproduction, development, and metabolism of aquatic organisms [32]. 

Most PhAC are currently not included in routine or regulatory control programs, which makes them candidates for future legislation. In this sense, the Commission Implementing Decision (EU) 2020/1161 has established a watch list of potential water pollutants for Union-wide monitoring in the field of water policy. Thus, azole pharmaceuticals, antibiotics and antidepressants such as clotrimazole, fluconazole, miconazole, amoxicillin, ciprofloxacin, sulfamethoxazole (SMX), trimethoprim and venlafaxine are some of the substances or groups of pharmaceuticals comprised [33].

The environmental concern related to the presence of PhAC in surface and groundwater is related to quantity, as well as their persistence and potential harm to human and aquatic life. The subsistence of trace pharmaceuticals and other contaminants in drinking water is a public health concern, since the potential chronic health effects associated with long term ingestion are scarcely known [34]. Although some compounds do not produce acute toxicity in the aquatic environment at low concentrations, constant release may lead to chronic and undesired synergistic effects with other compounds [4].

## 3. Enzymatic Biodegradation 

Enzymes are catalysts that conduct, within the mild conditions of temperature and pH, chemical reactions at a remarkably high rate, efficiency and specificity [22]. Several enzyme systems have been used for the efficient transformation and degradation of organic pollutants and have been shown to oxidize and degrade the pollutants into smaller intermediates [35]. 

The literature survey shows that most enzymatic remediation studies use oxidoreductase enzymes [36,37]. These enzymes are largely produced by the white-rot fungi (WRF), as extracellular enzymes for the degradation of lignin. WRF and their ligninolytic enzymes, namely lignin peroxidase (LiP), manganese peroxidase (MnP), versatile peroxidase (VP) and Lac, have been demonstrated to be capable of transforming a wide range of compounds. This ability is a result of the structural similarities of several micropollutants to lignin, as well as the fact that ligninolytic enzymes are substrate-nonspecific [38]. Other enzymes are, as well, used as biocatalysts for the remediation of toxic compounds, such as tyrosinase, horseradish peroxidase (HRP) and phenoloxidase [36].

Peroxidases (EC 1.11.1.X) are a vast group of heme-containing oxidoreductases, which use hydrogen peroxide as an electron-acceptor to catalyze oxidative reactions [39], whereas Lac (EC 1.10.3.2) are glycosylated multi-copper oxidases that catalyzes one-electron oxidation of various substrates associated with the reduction of molecular oxygen to water, via a radical-catalyzed reaction mechanism [40]. In contrast to peroxidases that require the supply of hydrogen peroxide, Lac only requests oxygen as the final electron acceptor for the oxidation reaction to occur, offering an alternative green approach for the biodegradation of several PhAC [41]. Lac is selective towards phenolic compounds, but due to the non-specificity, they are also able to degrade aromatic amines and related substances, thiol groups, diamines, N-heterocycles and phenothiazines [42].

**Figure 2:**
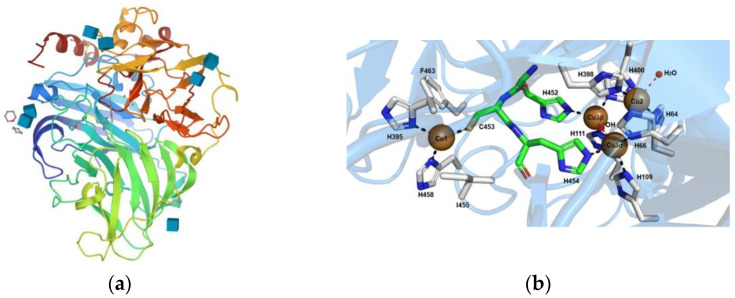
Lac from Trametes versicolor (Protein Data Bank code 1KYA) (**a**). Representation of the different amino acids of the catalytic site that coordinates the T1, T2 and T3 copper sites (**b**), reprinted from Ref. [46].

The active sites of Lac enzyme, as represented in Figure 2, contain four copper ions: one type 1 (T1) copper ion and a trinuclear copper cluster (TNC) composed of one type 2 (T2) copper ion coupled to binuclear type 3 (T3) copper ions [43]. The T1 copper site is the primary electron-acceptor for electrons offered by the substrate. The electrons are then moved to the TNC through highly conserved His-Cys-His tripeptide. After that, oxygen reacts with the fully reduced enzyme to form a peroxy-intermediate (PI), and then the PI is transformed back into a native intermediate, through a two-electron reduction. Once the fully reduced state is recovered, the final products are released from the TNC site. This results in the production of four radicals by the oxidation of four molecules of substrate, while one molecule of oxygen is reduced to two molecules of water and four protons (H^+^) are consumed from the solution [44,45].

The Lac and their active sites with copper ions play a key role in the reduction of oxygen. The redox potential (E°) difference between the T1 copper site and the substrate is one of the major factors that affect the oxidation rate. Accordingly, Lac are classified into three groups: low- (0.4–0.5 V), medium- (0.5–0.6 V) and high- (0.7–0.8 V) redox potential [47,48]. The variety of redox potentials is strictly related to the sources (e.g., bacterial, plant or fungi), due to the difference in the amino acid residues composition around the copper of the first reaction site. Lac from WRF has the highest redox potential, between 0.730 and 0.790 V, with phenylalanine as the non-coordinating axial ligand at the T1 copper site [49]. Among fungal species, Lac have been comprehensively identified from *Ascomycetes* and *Basidiomycetes*. Nonetheless, white-rot basidiomycetes such as *Trametes versicolor*, *Pleurotus ostreatus* or *Cerrena unicolor*, are noted for being efficient lignin degraders and Lac producers [36,50]. 

Several studies using enzyme extracts have been conducted for biodegradation of PhAC (Table 1). Although high efficiency rates are presented, the enzymatic wastewater treatment process is still relatively expensive, mainly due to the cost of commercial enzymes. One potential solution that may get the process cheaper is the application of crude enzymatic extracts. This might positively influence Lac catalytic activity and stability, as well as avoid the costly process of enzyme purification [39,51]. Furthermore, the crude extract offers efficient oxidation, since the synergistic action of the enzymes and a host of natural mediators and co-factors secreted by the fungi can potentially enhance the general performance [52]. 

A huge amount of renewable biomass is generated annually due to agricultural, food and industrial activities. Agricultural and food wastes are often disposed of indiscriminately or burnt off, thereby constituting environmental hazards and contributing to global warming through the generation of greenhouse gases. To achieve environmental sustainability, value-addition to wastes and promotion of advances in the circular economy, agro-food wastes are now being investigated for valorization through bio-enzymatic approach [63]. For fungal cultures, a wide variety of lignocellulosic biomass has been used as an alternative substrate in submerged fermentation, solid-state fermentation or semisolid-state fermentation. Peanut shell, wheat straw, wheat bran, rice straw, rice bran, agave bagasse, sugarcane vinasse, corn bran, fruit peel, tea, sunflower seeds and apple pomace are some of the studied lignocellulosic wastes for Lac production for water remediation [64,65,66,67,68,69,70,71,72,73]. 

Lonappan et al. [54] present a novel insight on residue valorization and found that apple pomace and pulp and paper solid waste were capable of inducing Lac production by *T. versicolor*. The resulting crude enzyme was effective in the degradation of diclofenac sodium (DCF) at an environmentally relevant concentration (0.5 mg/L). 

Kang et al. [61] stimulated the production of *Bjerkandera adusta TBB-03* Lac using lignocellulosic substrates as the sole enzyme inducer (e.g., ash wood chips). The formed Lac consistently showed a high capability to degrade acetaminophen (APAP) under various conditions. The authors also defended a 22% of improvement in SMX removal in the presence of APAP, that could act as mediator for oxidation. Enzyme suspension with lower purification levels may contain phenolic compounds that act as natural mediators and consequently increase removal efficiency. For instance, Lac from a mushroom substrate, based on sawdust and wheat bran, colonized by *P. ostreatus*, demonstrates significant removal of DCF (90%), bicalutamide (43%) lamotrigine (73%) and metformin (49%), when compared with the control with an uncolonized mushroom substrate [74].

Most Lac used to degrade pharmaceuticals to date are mainly sourced from fungi. However, heterologous expression of Lac genes has attracted increasing attention due to the requirement to find more specific enzymes tailored to the complexity of wastewater matrices [75]. Protein engineering may provide higher enzyme yields and may allow the production of Lac with improved properties such as operational stability, pH stability, solvent stability and thermostability [76,77]. For example, a novel Lac derived from *Bacillus tequilensis SN4* was demonstrated to be thermo-alkali-stable [77]. Lac Lcc9 from *Coprinopsis cinerea* expressed in *Pichia pastoris* also presented improved activity and stability at neutral and alkaline pH conditions [75]. Moreover, heterologous expressed bacterial Lac in *Escherichia coli* successfully degraded sulfanilamide antibiotics [78].

### 3.1. Laccase-Mediator Catalyzed System

Lac possess a relatively low redox potential (≤0.8 V) compared to peroxidase (>1 V). The redox potential difference between the substrate and the enzyme T1 copper affects the oxidative capacity of Lac. For example, Lac efficiently promotes single-electron oxidation of phenols. Accordingly, non-phenolic compounds, with redox potential above 1.3 V, are not directly prone to oxidation by Lac [79]. This lack of affinity of Lac is generally influenced by the distribution of functional groups in the chemical structure of the substrates. Compounds with electron withdrawing groups (EWG) such as carboxylic (-COOH), amide (-CONR_2_), halogen (-X) and nitro (-NO_2_) have lower enzyme affinity due to the less susceptible to oxidative catabolism [80].

The degradation efficiency of pollutants using Lac can be enhanced by the addition of redox mediators that are easily oxidized by the enzyme to free radicals. The presence of these small molecular weight compounds allows Lac to overcome a kinetic barrier and increase the spectrum of pollutants potentially degraded, as the mediator species have higher redox potentials. These compounds act as an “electron shuttle”, enabling the oxidation of complex substrates by highly reactive radicals that result from mediator oxidation by Lac. These radicals may return to their parent compound through reduction during the oxidation of the target pollutant [52,81].

Table 2 presents a list of redox mediators and their related information. The hydrogen atom transfer (HAT), electron transfer (ET) and ionic mechanisms are the primary mechanisms for mediator oxidation of a compound. The oxidation mechanism of the 2,2′-azino-bis (3-ethylbenzothiazoline-6-sulfonic acid) diammonium salt (ABTS) and 2, 2, 6, 6-tetramethyl-1-piperidinyloxy (TEMPO) radicals follows ET and ionic mechanisms, respectively. As well as *p*-coumaric acid, hydroxybenzotriazole (HBT), N-hydroxyphthalimide (HPI) and syringaldehyde (SA) follow HAT oxidation mechanism [18]. 

The mediator type and concentration have a major impact on the practicality and feasibility of using Lac in PhAC degradation. Some literature studies that used mediators coupled with enzymes for pharmaceutical transformation are presented in Table 3. Naproxen (NPX) is an example of a recalcitrant drug able to resist enzymatic oxidation due to the presence of the EWG carboxyl and the absence of any strong electron-donating groups (EDG). Lac mediated with HBT achieved the greatest removal of NPX (80%) at the highest mediator concentration of 1 mM, while the performance of violuric acid (VLA), another N-OH type mediator, was weaker (~60%). At an inferior dose (0.5 mM), HBT and ABTS mediators still presented very efficient removal rates of NPX [82].

Low molecular weight mediators allow complex compounds to access the active site of the enzyme. The oxidation of HBT by Lac generates small aminoxyl radicals that can remove the H atom from the O-H bond of phenolic substrates and consequently can create the phenoxyl radicals. The aforementioned mechanism may be responsible for the significant improvement in the degradation of salicylic acid [86]. 

In contrast to the low efficiency of the Lac degradation process without mediators, Lac coupled with ABTS revealed complete degradation of tetracycline (TC) and oxytetracycline (OTC) after only 4 min and 5 min, respectively [41]. Naghdi and co-authors [56] observed that 95% removal of carbamazepine (CBZ) was possible due to the combination of ABTS with crude Lac from *T. versicolor*. Furthermore, they reported that the enzyme concentration had a quadratic effect on biotransformation since an optimum level of Lac activity led to a rapid generation of ABTS radicals and caused an efficient transformation of CBZ, but further addition of enzyme to the solution increased the collisions and interactions and could block enzymes active sites. In the case of Lac-catalyzed degradation of the antibiotic chloramphenicol (CAP), Navada and co-authors [51] concluded that natural mediators (SA and vanillin) exhibited lower K_m_ values than the reactions mediated by the synthetic mediators (ABTS and α-naphthol), showing that the natural mediators have a higher affinity to Lac than CAP and, therefore, increased the rate of oxidation reactions.

**Figure 3:**
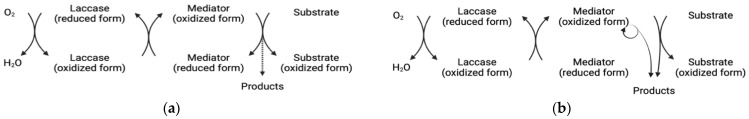
Ideal Lac-mediator reaction model (**a**). Alternative Lac-mediator reaction model (**b**). Adapted from Ref. [81], with permission from Elsevier. License number: 5394140329531.

Ideally, during the oxidation of the pollutant, only oxygen is consumed in the catalytic cycle. However, the consumption of the mediator during the reaction is also possible (Figure 3a). In this case, “Lac enhancer” is a more accurate term. Margot and co-authors [81] investigated and showed the potential of the Lac-mediator system for the degradation of the antibiotic SMX with ABTS, SA and acetosyringone (AS). From their results, neither ABTS, SA nor AS acted as catalysts, since these three mediators were consumed during the reaction, with a mediator/pollutant molar ratio between 1.1 and 16. The authors proposed an alternative model of oxidation in which Lac oxidize mediators to reactive radicals, that can transform into more stable products and react with each other or with pollutants (Figure 3b). 

Bankole and co-authors [84] demonstrated the effectiveness of ABTS, HBT and *p*-coumaric acid as redox mediators in the degradation of olsalazine by Lac from *Aspergillus aculeatus*. Furthermore, they observed that the increase in degradation efficiency was proportional to an increase in the concentration of mediators till a threshold value is reached, and at this point, no significant enhanced degradation of the pharmaceuticals occurs. This result is in line with the general trend observed in the literature that such threshold concentrations depend on the source of Lac, the target compound and the mediator used [52]. 

On the other hand, Lac catalyzed reaction with TEMPO, which produces the oxoammonium cation, was able to greatly promote atenolol (ATL) transformation in an aqueous solution, with complete removal after 12 h [85]. However, Wang et al. [85] also found that Lac would be deactivated more rapidly in the reactions with higher mediator concentration as a result of distortion or blockage of enzyme active sites by radicals. Other findings show that free radicals produced by the oxidation of a mediator can destabilize the enzyme by reacting with the aromatic amino residues on its outer surfaces [82].

Catalytic degradation using ligninolytic enzymes such as Lac with redox mediators may represent an alternative clean strategy for PhAC removal from the water matrix. However, the viability of this solution in real treatment systems is limited due to the necessity for high concentrations of mediator and the formation of several remediation products in concentrations eventually higher than the original pollutant [81].

### 3.2. Transformation Mechanisms and Toxicity Evaluation

Enzymes transform complex compounds into simpler substances and it is unknown whether pharmaceuticals are metabolized by remediating enzymes to less or more toxic products. There is also a lack of knowledge on the structure of the metabolites resulting from pharmaceuticals degradation process. Therefore, some authors have studied the transformation pathways, as well as metabolites toxicity or estrogenic activity. 

Considering molecular weight and chemical structure, Kózka et al. [87] observed three main types of transformations of a series of antidepressants and immunosuppressants carried out by fungal ligninolytic enzymes. The first one is chemical oxidation and occurred for clomipramine, mianserin, sertraline, fluoxetine and citalopram. The second transformation is straight demethylation or demethylation coupled with other reactions such as oxidation or deamination, and was observed for clomipramine, mianserin, sertraline and venlafaxine transformation. The third type of transformation is the oxidative cleavage of the molecule into two parts of comparable size and was observed for fluoxetine and paroxetine. 

Kasonga et al. [88] proposed the metabolic pathways for ibuprofen (IBP) and CBZ based on detected intermediates by a fungal consortium of Lac, LiP and MnP. The IBP transformation pathway appeared to result from hydroxylation with addition of hydroxyl group to 1,2-dihydroxy-IBP or carboxylation reaction leading to the substitution of the methyl group by carboxylic group to form IBP carboxylic acid. Furthermore, the CBZ metabolic pathway was presented in four routes. The first route proposed was oxidation or hydrolysis to iminostilbene. The second route consisted of oxidation reactions of the carbons on the aromatic benzene group to CBZ-2,3-quinone. The third route combined hydroxylation, hydrolysis and then oxidization to iminoquinone. The fourth and principal metabolic route started with oxidation or epoxidation and the end products were acridone, 10,11-dihydro-10-hydroxy-CBZ and 9-hydroxymethyl-10-carbamoyl acridan. Likewise, according to Naghdi et al. [56], 10,11-dihydro-10,11-dihydroxy-CBZ and 10,11-dihydro-10,11-epoxy-CBZ are considered to be the primary metabolites from CBZ oxidation by Lac-ABTS. Moreover, toxicity tests revealed that these products had no estrogenic effect.

In another study related to the degradation of DCF by Lac, the authors identified 3′-hydroxydiclofenac, 4′-hydroxydiclofenac, and 5-hydroxydiclofenac as the major transformation products [54].

Yang et al. [89] demonstrated that immobilized *C. unicolor* Lac was effective in detoxification of TC antibiotics and identified three transformation products with LC-TOF-MS. According to the authors, TC is first oxidized by Lac to the corresponding ketone and then the amino group is bi-demethylated to form the second transformation product. The final product results from oxidation, followed by water elimination and dehydrogenation. 

Lac oxidation of SMX in presence of ABTS possibly results in two products from pharmaceutical degradation and a third one from ABTS oxidation by Lac, designated 3-ethyl-6-sulfonate benzothiazolinone imine [81]. In another study, Tian et al. [41] demonstrated that TC was first oxidized by Lac-ABTS to OTC as the major transformation product and proposed a degradation pathway including deamination, demethylation and dehydration. 

The assessment of the overall toxicity of the treated effluents is essential to provide a more complete outlook of the environmental relevance of the enzymatic treatments in real practical applications. Several metabolites result from enzymatic water treatment and additional toxicological information by means of bioassays is imperative. These studies are able to describe the ecotoxicity and the estrogenic activity of the resulting metabolites. 

Presently, there are examples of pharmaceutical enzymatic degradation that produce fewer toxic compounds and lack of estrogenicity effect. A micro-toxicity study with *Pseudokirchneriella subcapitata*, *Candida albicans*, *Cryptococcus neoformans* and *Saccharomyces cerevisiae* revealed that Lac-HBT treated ketoconazole and its isolated metabolites, such as 1-(4-{4-[2-(2,4-dichloro-phenyl)-2-imidazol-1-ylmethyl-[1,3]dioxolan-4-ylmethoxy]-phenyl}-4-oxy-piperazin-1-yl)-ethanone, suffered a decrease in the toxicity levels. The presence of the oxygen atom in the structure of metabolites reduces their lipophilicity and decreases their toxicity [83]. Furthermore, Lac-SA mediated system led to the transformation of CAP in chloramphenicol aldehyde and had less toxicity for microbial growth than mediators vanillin, ABTS and α-naphthol [51].

Spina et al. [90] showed that crude Lac from *Trametes pubescens* MUT 2400 was very active against all target micropollutants as ketoprofen, present in real municipal wastewater. Estrogenic analysis and toxicological tests with *Raphidocelis subcapitata* and *Lepidium sativum* showed a clear ecotoxicity reduction of treated wastewater. Similarly, Sun et al. [50] reported that a concentrated Lac isolated from *Trametes hirsuta* was capable of effectively metabolize 17b-estradiol (E2) more than 99% and potentially lead to a reduction of the estrogenic activity of E2. Through the combination of 13C-isotope labelling with high-resolution mass spectrometry, the dimers, trimers and tetramers were recognized as the primary by-products of E2 metabolism.

Contrarily, Becker et al. [91] verified that Lac mediated with SA effectively removes (>50%) a broad range of antibiotics after 24 h. However, this enhanced degradation induces unspecific toxicity. Furthermore, Feng et al. [85] verified that the transformation of ATL via Lac/TEMPO-catalyzed reaction greatly reduced the mortalities of zebrafish (Danio rerio) eggs, but the degradation products and the residual TEMPO still possess toxicity (approximately 40%). This transformation mainly involved hydroxylation, carbonylation, C–O bond cleavage and coupling reactions.

### 3.3. Immobilized Biocatalytic System

The practical application of freely suspended enzymes exhibits high activity in the biotransformation processes. Nevertheless, the free form is limited by the low stability and high cost of production for large scale implementation due to the impossibility of recovery [92,93]. To overcome such limitations, several studies have already demonstrated that enzyme activity and stability can be improved by immobilization on solid supports [36]. 

The stabilization of the peptide structure of the biocatalyst, creating interactions between the enzyme and an immobilization matrix, leads to enzyme stability and resistance improvement towards extreme operational conditions, including strong pH, high temperature or the presence of organic solvents [94]. Furthermore, immobilization allows the easy recovery of the enzyme and offers high reusability in several catalytic cycles without significant loss of its unique properties, which reduces operating costs [95]. 

The immobilization strategies are divided into methods based on physical or chemical interactions between enzymes and supports [96]. Physical immobilization involves the creation of non-specific interactions via hydrogen bonds, ionic and hydrophobic interactions. The physical methods include entrapment, encapsulation and adsorption, and there is no requirement for the functionalization of the support [36]. On the other hand, chemical immobilization includes enzyme attachment to the matrix by covalent binding or cross-linking [97]. An example of a covalent binding agent is glutaraldehyde that is capable to react with the amine groups at the surface of both enzymes and support through the formation of Schiff’s bases and Michael’s adducts [98]. Moreover, enzymes can be cross-linked to each other or create a cross-linked enzyme aggregate (CLEA) [99].

Physical adsorption is simpler and leads to higher final enzyme activity. However, desorption or leakage of the immobilized enzyme is common with cycles of use due to the relatively weak binding forces. Oppositely, chemical immobilization leads to partial deformations in the enzyme molecular shape but offers a robust attachment of enzymes to the support [94,100]. The characteristics of the support are essential to define the success of the final biocatalyst, therefore the ideal support for usage of industrial applications should be inert, rigid, inexpensive, eco-friendly and present thermal and mechanical resistances [24]. The support matrices can be classified according to their chemical composition as inorganic materials, organic materials, hybrids and composite materials. A large diversity of support has been developed due to the search for better stability and scale-up performance. Several recent methods of enzyme immobilization are summarized in Table 4. 

A wide range of materials are used for enzyme immobilization. Zdarta and co-authors [93] studied Lac immobilization by adsorption and encapsulation using poly(l-lactic acid)-co-poly(ε-caprolactone) (PLCL) electrospun nanofibers. After 24 h, encapsulated Lac biodegraded over 90% of NPX and DFC, contrasting with an adsorbed enzyme which presented lower removal efficiency, 60% for NPX and 80% for DFC. This is mainly justified by the deactivation and elution of enzymes from the support. Dong and co-authors [110] described a mediating system in which Lac was assembled, over π-π interactions, onto pristine few-layer graphene (FLG) surface. The composite effectively transformed beta-blocker labetalol for more than 10 cycles, as the FLG increases the exposure extent of the catalytic center with the enhancement of the catalytic activity. 

Immobilization of enzymes provides protection against denaturation and conformational changes. For example, Sharifi-Bonab and co-authors [23] immobilized Lac on graphene oxide (GO) nanosheets, followed by entrapment in alginate biopolymer. The immobilized Lac retained more than 70% of its initial activity after 10 days, contrarily to free Lac that became inactive. Furthermore, according to Zdarta and co-authors [107], Lac immobilized in mesostructured cellular foam (MCF) silica retained higher activity (80%) over a wider range of temperature and pH. The enzyme immobilized onto MCF + Cu preserved almost 90% of its initial activity after 10 cycles since the MCF has a protective effect towards Lac molecules immobilized onto the surface and into its pores. Similarly, Lac can enter into the narrow mesopores of meso-MIL-53(Al) by undergoing conformational changes and becoming immobilized in the mesopore-MIL-53(Al), thus the entrapment force is significantly higher compared to conventional physical adsorption [111]. In another example, Nguyen and co-authors [96] immobilized Lac on acid-washed granular activated carbon (GAC) via physical adsorption and observed that GAC-bound Lac maintained full activity for up to 8 cycles of continuous application.

Covalent binding of enzymes on solid materials for pollutant removal has been intensively investigated and yields efficient results. Masjoudi and co-authors [94] described a covalent immobilization of Lac on polyvinylidene fluoride (PVDF) membrane modified with multi-walled carbon nanotubes (MWCNT) and observed successfully removal of DCF (95% in 4 h) in a mini-membrane reactor. Likewise, Maryšková and co-authors [105] immobilized the Lac onto polyamide/polyethylenimine (PA/PEI) nanofibers, via covalent attachment, with the Lac retaining more than 52% of initial activity after 30 days and successfully degrading triclosan (~70%) and 17α-ethynylestradiol (~50%) in real wastewater effluent. In another work, Lac immobilization on titania nanoparticles (TiO_2_), with the functionalizing agent 3-aminopropyltriethoxysilane (APTES) and glutaraldehyde cross-linker, showed remarkable stability at 50 °C and 60 °C and low pH values of 2 and 3 [101]. 

Compared with literature data, Apriceno and co-authors [49] reported that a quite low value of enzymatic activity (0.02 U of enzyme) was required for 90% of degradation of DCF (50 mg/mL) in 3 h, when the Lac was covalently immobilized on chitosan beads and coupled with ABTS. Yaohua and co-authors [108] also reported an efficient degradation of índole, even after 5 and 10 cycles, using a co-immobilization method, which consisted on the encapsulation of ABTS molecules into the dual-functionalized cellulose beads, followed by covalent binding of Lac.

Magnetic cross-linked enzyme aggregates (M-CLEA) are another example of the enzyme immobilization method, in which amino-functionalized magnetic nanoparticles are used. Yang and co-authors reported that Lac immobilized as M-CLEA eliminated over 80 µg/mL of TC in 12 h [89]. Similarly, CLEA and M-CLEA Lac showed high DCF removal capacity (~80%) at 1 and 5 µg/L pollutant concentrations [99].

The use of immobilized biocatalysts for PhAC transformation leads to sustainable industrial process performance. Despite the promising results, there are still issues to be further investigated such as the production costs of the immobilized enzymes, the possibility of scaling biocatalytic systems, as well as storage stability and the treatment potential of numerous PhAC in real wastewater effluents using enzyme immobilized systems. 

## 4. Factors Affecting the Enzymatic Degradation

Enzymatic treatment of recalcitrant PhAC may be affected by several reaction conditions, e.g., temperature, pH and natural organic matter. The enzymes are mainly stabilized by weak interactions such as Van der Waals and hydrogen bonds, and the latter is largely influenced by the medium pH [112]. The relationship between the optimal pH and Lac activity is also dependent on the substrate and the redox potential difference between the substrate and the enzyme T1 copper site. Enzyme activity of Lac may decrease at higher pH because of the formation of hydroxide anion, which disturbs/blocks the internal electron transfer from T1 to T2/T3 copper in Lac, due to the attachment of hydroxide anion in the T2/T3 coppers [21,113]. Lac from *T. versicolor* is not able to degrade doxorubicin (DOX) at pH 3, but by increasing the pH to 4 the compound starts to be degraded. Therefore, increasing the pH the degradation also increases until the neutrality [21].

Temperature plays an important role in the rate of biological reactions, therefore the lower degradation efficiency at inferior temperature (e.g., 25 °C) is related to a reduced activation energy of the reaction, whereas above a certain temperature the enzyme can be inactivated due to denaturation [56,113]. An almost complete removal of CBZ by the Lac-ABTS system is observed at pH 6 and 35 °C, while the degradation efficiency varies from 69% to 73% at 25 °C and 45 °C, respectively [56]. Lonappan and co-authors [54] found that the maximum degradation of DCF by Lac from *T. versicolor* occurred at pH ranging from 4 to 5 and a temperature of 50 °C. However, the optimum removal of ketoconazole with Lac is achieved at pH 4.5 and 45 °C in 6 h [83].

As mentioned, the reactivity of Lac is influenced by the presence of EDG and EWG. According to Yang and co-authors [80], EDG such as hydroxyl (-OH), amine (-NH_2_), alkoxy (-OR), alkyl (-R) and acyl (-COR) groups were susceptible to electrophilic attack by oxidase enzymes. Conversely, the presence of EWG reduced the affinity of enzymes. Rodríguez-Delgado and co-authors [55] described higher biotransformation (78%) of the antibiotic 5,7-diiodo-8-hydroxyquinoline (DHQ), which despite having an electron withdrawing iodine halogens group in its structure, also contains a strong electron-donor hydroxyl group that enables Lac oxidation. 

The presence of ions in aqueous media may result in different effects on the catalytic performance of the enzyme. For example, halide anions (F, Cl and Br) and hydroxide anion (OH) possibly bind to the T2 copper of Lac and interrupt the internal electron transfer between T1 and T2/T3, as well as they can bind near the T1 active site and block substrate access. Lac from *T. versicolor* exhibited 20% of activity inhibition to 5 mM sodium chloride, a usual concentration of this compound in municipal wastewater [114]. Cu^2+^-assisted Lac enhanced the transformation of triclosan as well, possibly due to the production of more phenoxy radicals. Conversely, Mn^2+^ revealed a thorough inhibition on the transformation of triclosan, since it can be associated with the formation of the Mn^3+^-citrate complex which results in oxygen consumption and interruption of electron transport in reaction systems [62]. Tian et al. [41] revealed that the presence of the Mn^2+^ ion inhibited the removal rate of TC and OTC by the Lac-Q–ABTS system (*Pycnoporus* sp. *SYBC-L10*), while the presence of Al^3+^, Cu^2+^, and Fe^3+^ accelerated the removal rate. 

Humic acids (HA), as common substances derived from the organic matter decomposition, often appear in aquatic environment, and influence the Lac-catalyzed reaction. The presence of HA (0–20 mg/L) inhibited ATL transformation by TEMPO-mediated Lac, through competition reaction with the enzyme. The same effect holds for inorganic anions, HCO_3_^−^ and CO_3_^2−^, that can react with the reactive radicals (e.g., SO_4_^•−^ and HO^•^) and affect the transformation of target pollutants by changing solution pH [85].

## 5. Bioreactors 

Bio-catalysis is a reliable tool for developing green and strengthened processes, as long as the proper reactor configurations are combined. The selection of the reactor configuration and the operation strategy is directly related to the kinetic behavior and characteristics of the enzyme and/or support in immobilized systems [115]. Different reactor configurations have been studied for the catalytic treatment of PhAC, such as stirred tank reactor, fluidized bed bioreactor, packed-bed reactor and enzymatic membrane reactor (EMR) [96,99,116,117]. The typical configuration of an enzymatic reactor is a batch system, which is flexible, simple and provides easy control of both temperature and pH of biodegradation. However, the implementation of continuous flow reactors is essential to attain a stable quality of the effluent, high productivity and low operational costs [118]. 

Under continuous operating conditions, Lonappan et al. [119] assessed the degradation of DCF at environmental concentrations by Lac bound to pine wood and pig manure biochar, in a fixed-bed column. The combination of adsorption and catalytic effect resulted in more than 70% removal of DCF. Similarly, Nguyen and co-authors [96] showed that GAC-bound Lac in a packed-bed column efficiently removed SMX, CBZ, DCF, due to adsorption on the carrier and degradation by the enzyme, during continuous operation over two months with a throughput of 12,000-bed volumes.

Some adverse effects in strategies that directly use the enzyme-producing fungus such as bacterial contamination, are felt during the continuous process and may further present a negative impact on enzymatic degradation. For example, Li et al. [120] reported that in a fixed-bed bioreactor packed with a mixture of WRF mycelia pellets, the removal efficiencies for CBZ and NPX dropped from 60% and 95%, respectively, to less than 20% after 14 days, possibly due to the contamination by other microorganisms in the reactor. Thus, a treatment with sodium hypochlorite was forwarded as solution, which led to an increase in the removal of NPX by more than 90%. 

Torán and co-authors [121] studied a treatment that guarantees fungal stability under continuous treatment conditions. High removals in real hospital wastewater spiked with PhAC were obtained in a trickling packed bed reactor using *T. versicolor* immobilized on pallet wood, during 49 days. Comparing with fluidized bed reactor, excessive bacterial contamination was avoided by limiting the nutrient supply and controlling the pH. In another study, Li and co-authors [122] employed a rotating suspension cartridge reactor immobilized with *Phanerochaete chrysosporium* to treat synthetic wastewater for 160 days under non-sterile conditions. The strategies of immobilization of fungi on foam cubes, the pattern of liquid/airflow inside the cartridge created by intermittent operational mode and the gradual cut of the external carbon source, allowed feeding the cartridge repeatedly and also caused the timely washed off of aging microorganisms.

Only a few studies have dealt with the removal of pharmaceuticals from real wastewater. For example, Tormo-Budowski and co-authors [123] reported that a fungal recirculating trickle-bed bioreactor was able to remove pharmaceutical compounds to a great extent from synthetic (89%) and real wastewater (90%), mostly due to adsorption to the bed’s biomass. Acute toxicity tests showed an additional decrease in wastewater toxicity when compared to an identical study with a semi-batch stirred tank bioreactor. 

Most of the work for treating wastewater matrices has been done on a small scale, which subsequently should be scaled up gradually to determine the system performance in large WWTP.

## 6. Enzymatic Membrane Reactor (EMR)

Enzymatic degradation of pollutants is mainly investigated in batch bioreactors due to the concern of enzyme washout along with the treated effluent as in a continuous flow bioreactor [124]. To overcome this drawback, biocatalysts can be immobilized onto a large variety of carriers, as well as in membranes of suitable molecular weight cut-off coupling with an enzymatic bioreactor. The EMR consists of coupling a membrane separation process with an enzymatic reaction, in which a semi-permeable membrane promotes the separation of the enzyme from products and/or substrates and creates a selective physical/chemical barrier. 

EMR enables the retention and reuse of the enzyme once the end products have been recovered in the permeate. For example, De Cazes and co-authors [125] used a ceramic membrane to retain Lac and their EMR was able to reach in batch mode a degradation rate of 0.34 mg of TC per hour during 10 days. Therefore, this approach offers several advantages over other alternatives, namely: (i) more effective retention of enzyme, (ii) enzyme operation that avoids limiting mass transfer associated with immobilization on carriers and (iii) easy enzyme replenish during long term operation [117]. 

The EMR system is constituted by a membrane that acts as a selective barrier and the enzymatic degradation reaction takes place during the mass transfer process [18]. Recent studies have explored ultrafiltration enzymatic membrane reactors (UF-EMR) due to their potential to retain the enzyme and the continuous removal of micropollutants. In this regard, Nguyen and co-authors [126] presented a methodology based on the concentration of crude Lac using an ultrafiltration (UF) membrane before its application in EMR. During the filtration process, an enzymatic layer is retained and can later adsorb and oxidize the substrates. Overall, the authors described that pharmaceutical compounds such as amitriptyline, salicylic acid, triclosan and gemfibrozil were effectively removed. Asif and co-authors [124] compared the performance of UF and nanofiltration (NF) combined with EMBR for the degradation of atrazine, CBZ, SMX, DCF and oxybenzone. Mass balance analysis revealed that micropollutants degradation was improved by 15–30% in NF-EMBR as compared to UF-EMBR, which can be attributed to the prolonged contact time between Lac and substrates following their effective retention. 

**Figure 4:**
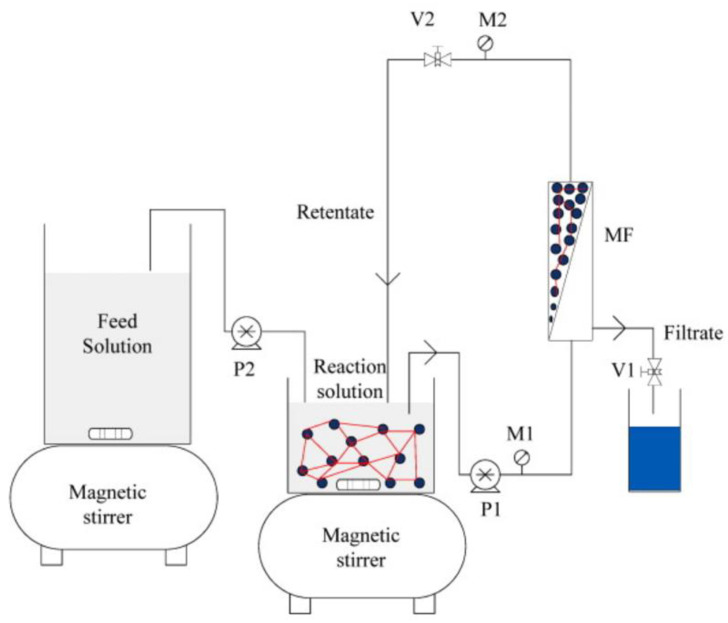
Schematic representation of the HBR experimental set-up. Reprinted from Ref. [128], with permission from Elsevier. License Number: 5394210088431.

UF and NF membranes, with their minuscule pore sizes, are effective in removing micropollutants. However, during a continuous process, membranes present a drop in permeate flux due to membrane fouling under high organic matter content. This requires periodic membrane flushing and constitutes a potential challenge for large scales applicability [127]. In this sense, Ba and co-authors [128] proposed a cost-effective hybrid bioreactor (HBR) by combining microfiltration membrane (MF) with CLEA-Lac to remove PhAC (Figure 4). This system proved to limit high concentrations of natural organic matter within the membrane and prevent its rapid fouling, while the biocatalyst was confined within the MF membrane and recycled back into continuous operation. In another study with HBR, which combined the synergistic action of crosslinked tyrosinase and Lac aggregates with hollow fiber MF, the authors reported 90% efficiency for the elimination of 14 selected PhAC from municipal wastewater at an environmentally relevant concentration of 10 mg/L [129].

## 7. Real Wastewater and Scale-Up

Chemical and biological complexity of wastewater may strongly interfere with enzymatic activity. Spina et al. [90] evaluated the stability and the activity of *T. pubescens* Lac during the transformation of micropollutants present in primary sedimentation (W1) and at the end of the process (W2) of municipal wastewater. During the 24 h experiment, enzymatic activity was strongly inhibited by the complex matrix and W1 showed considerable destabilizing potential, due to a more developed microbial community and higher load of suspended solids. However, these enzymes were very active against all the target micropollutants and caused a significant abatement of the potential ecological impact. In another study, the authors used Lac as a treatment on municipal wastewater from WWTP, comprising PhAC, pesticides, plasticizers. Although enzyme stability was impaired by the composition of the effluent matrix, transformation above 70% was achieved for most micropollutants during 24 h with a decrease estrogenic activity [57]. 

The application of biocatalysts in industrial and continuous bioremediation processes is limited. The free enzymes are susceptible to inactivation over time due to unfavorable conditions, weak retention and reusability of the biocatalyst. Furthermore, most studies with real wastewater have investigated the removal of PhAC by immobilized enzymes. For example, immobilized Lac from *T. versicolor* and *Myceliophthora thermophila* can effectively remove endocrine activity of mixtures of EDC in wastewater, with high removal rates for estrogenic (82% removal after 24 h) and androgenic activities (99% removal after 6 h) [130]. Additionally, Arca-Ramos et al. [131] studied the stability of free Lac and magnetically separable M-CLEA in secondary effluent collected from the municipal WWTP (Magog, Canada). The M-CLEA showed higher stability against inhibitors, acidic pH and wastewater matrix, as well as showed the ability to transform the phenolic compound APAP and certain non-phenolic PhAC, as mefenamic acid, fenofibrate and indomethacin, with similar or even higher efficiency than free Lac. Le et al. [132] established that Lac encapsulated in an alginate matrix exhibited satisfactory performance under extreme environmental conditions in the presence of metal ions or other components. The encapsulation method protected enzymes from environmental factors that inhibit enzymatic activity during practical applications, particularly for wastewater treatment.

For the abatement of PhAC from the effluents of WWTP, several approaches using ligninolytic fungi have been tested. *P. ostreatus* and its spent mushroom substrate were combined to produce a biofilter for the removal of sulfonamides from real water matrices. The fungi activity coupled with the adsorption capacity of the biofilter showed an effective removal rate around 100% in 24 h [133]. Likewise, Křesinov et al. [134] employed *P. ostreatus* as a tertiary treatment in a WWTP to remove endocrine disruptors and achieved removal rates of 76% in a pilot-scale trickle-bed reactor within 24 h.

One of the main sources of PhAC is hospital wastewater (HWW). Fungal consortium treatments can remove these pollutants from real wastewater, but fungal survival can be affected by bacterial competition. Mir-Tutusaus et al. [135] reported that *T. versicolor* was able to treat real non-sterile HWW in a continuous fungal fluidized bed bioreactor for 56 days after a coagulation-flocculation pretreatment. The fungal operation removed 90% of the initial concentration of antibiotics (5000 ng/L), but gradually lost the removal capacity to values around 50%. In the case of psychiatric drugs, removals of about 50% of the initial load was reached. In another study, Ferrando-Climent et al. [136] evaluated the oxidative enzymatic system of *T. versicolor* to eliminate anticancer drugs from HWW. Ciprofloxacin was eliminated more efficiently at non-sterile conditions, which can be attributed to a synergistic degradation contribution of fungi and common fecal bacteria. In the case of tamoxifen, higher removal was obtained under sterile conditions by combined sorption-biodegradation processes. 

Biocatalytic efficiency can be increased by adding McIlvaine’s buffer to real wastewater effluents, by providing beneficial anions that stabilize enzymes and thus enabling their activity [137]. Despite the promising results already presented in the literature, enzymatic treatment needs to be established for micropollutants in real wastewater, as the study in aqueous matrices, such as pure buffer or deionized water, can be a source of misleading or impractical data.

## 8. Hybrid Methods

The study of combined processes for the removal of organic micropollutants is of great importance. The partially transformed intermediates of a pollutant may have high reactivity in some other processes and the interaction between degradation processes may significantly improve the overall degradation rate and the final reaction products toxicity. Therefore, the cooperation of enzyme technology with other treatment technologies can be a valuable strategy for wastewater treatment.

**Figure 5:**
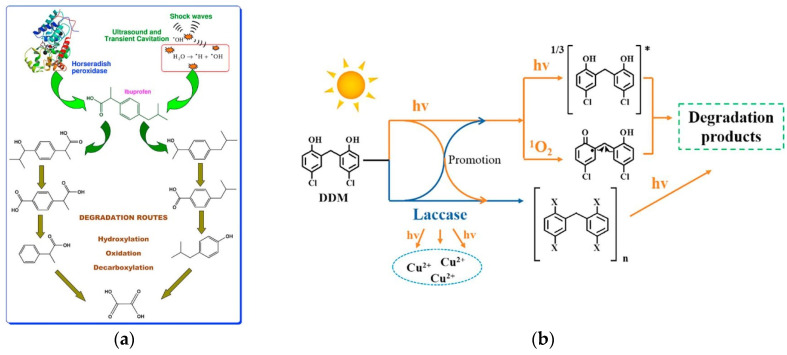
Degradation of IBP by sono-enzymatic treatment (**a**). Degradation of DDM by photolysis and Lac-catalysis in aqueous solution under simulated sunlight irradiation (**b**). Reprinted from Refs. [138,141], with permission from Elsevier. License Number: 5394210289329 and 5394210418986.

A novel sono-hybrid technique for the degradation of recalcitrant organic pollutants involves a sono-enzymatic treatment, in which sonolysis is combined with enzymatic treatment with peroxidase enzymes such as LiP, MnP and HRP. Chakma et al. [138] reported the usage of sono-enzymatic degradation of IBP using the HRP enzyme. At low ultrasound frequency and static pressure, sono-enzymatic treatment was revealed to be more effective than the individual techniques. A positive synergy was observed due to the formation of hydrophilic intermediates induced by radicals from transient cavitation that were rapidly degraded by the enzyme (Figure 5a). In another study, authors demonstrated that Lac from *T. Versicolor* (0.5 U/L) combined with ultrasonication removed over 60% of chlortetracycline (2 mg) spiked in wastewater in 2 h, whereas Lac treatment alone took 2 days to degrade 87% of it [64]. Similarly, Sutar et al. [139] reported a higher degradation in reduced time using ultrasound-assisted enzymatic degradation for ciprofloxacin hydrochloride, as compared to the conventional method.

In the study conducted by Vasiliadou et al. [140] advanced bio-oxidation systems based on WRF (*T. versicolor* and *G. lucidum*) in the presence of quinone-type mediators were able to induce the production of highly oxidizing hydroxyl radicals to degrade recalcitrant pollutants from wastewater such as pharmaceuticals. The application of 2,6-dimethoxy-1,4-benzoquinone (DMBQ) in the redox cycling process of fungi resulted in 60–100% removal of 13 pharmaceuticals due to the high affinity of Lac enzyme for DMBQH_2_. Another publication presented by Shi et al. [141] demonstrated that the combination of photolysis and Lac-catalysis in an aqueous solution under simulated sunlight irradiation could synergistically promote the dichlorophen (DDM) removal. Results demonstrated that photolysis could efficiently remove the harmful coupling products generated in the Lac-catalysis of DDM (Figure 5b). In another application, Zhu et al. [142] reported that activated sludge treatment coupled with chloroperoxidase improved the removal of lincomycin.

## 9. Concluding Remarks and Prospects

The presence of pharmaceutical micropollutants in wastewater and the consequent unknown risks of their concentration as well as synergistic or antagonistic interactions between them increases the demand for environmentally friendly and economically viable processes to reduce their environmental impact. As previously summarized, enzyme-based degradation of contaminants in wastewater is emerging and several studies have been conducted using oxidoreductases, predominantly Lac, as these enzymes have an enormous potential for pharmaceutical degradation and wastewater remediation. 

Enzymatic wastewater treatment has shown its feasibility in pharmaceuticals removal. However, this degradation approach still has some limitations that prevent its use in large-scale set-ups. Briefly, the current challenges of enzymatic treatment focus on yields, efficiency and the high costs of enzymes. Although the studies presented are quite promising, it is possible that in some cases certain remediating enzymes might eventually produce intermediates eventually more toxic than the initial pollutant. Therefore, the transformation metabolites, as well as their toxicity or estrogenic activity, should always be assessed. In addition, this technology may need to be combined with other methods to achieve complete remediation, since enzymes only transform complex compounds into simpler substances but further steps may be needed to tackle these last ones.

The enzyme free form systems can achieve good treatment performance, but present low stability and high loss of enzyme during the treatment. The immobilization strategy increases the catalyst stability and enables its reusability, which constitutes a more cost-effective and eco-friendly process. Thus, enzyme immobilization systems also grant the applicability of enzymatic bioremediation to an industrial scale process. However, most immobilization methods have significant drawbacks due to the still short enzyme lifetime that makes the process highly expensive, loss of enzyme activity and regeneration problems. Consequently, further research should be focused on the development of highly effective and safe treatment pathways with increased metabolic properties, enhanced recovery of enzyme activity, reusability during the immobilization process and inexpensive support options.

The application of the enzymatic approach to the treatment of real wastewater samples further comprises a potential limitation, since enzymes may suffer inhibition or denaturation in consequence of harsh conditions or the presence of natural organic matter and/or ions. Many studies were conducted under laboratory conditions using synthetic or spiked wastewater and mainly focused on the removal of limited compounds, usually in high concentrations, that do not represent realistic environmental situations. Thus, operational parameters should be tested under real-life conditions in order to move to pilot-scale and larger-scale systems. 

## Figures and Tables

**Table 1:** Examples of biodegradation of pharmaceutical micropollutants by enzymes.

Compound	Enzyme	Source	PhAC (mg/L)	Reaction Conditions	Enzyme Load (U/L)	Efficiency (%)	Ref.
Diclofenac	LiP	*Phanerochaete chrysosporium*	5	pH 4, 24 mg/L H_2_O_2_, 25 °C, 2 h.	180	100	[53]
	Lac	*Trametes versicolor*	1	pH 6.5, 25 °C, 5 h.	500	>90	[54]
	Lac	*Pycnoporus sanguineus*	100	pH 5, 25 °C, 8 h.	100	50	[55]
5,7-Diiodo-8-hydroxyquinoline	Lac	*Pycnoporus sanguineus*	100	pH 5, 25 °C, 3.5 h.	100	78	[55]
Carbamazepine	Lac	*Trametes versicolor*	1	pH 6, 35 °C, 24 h.	60	30	[56]
Salicylic acid	Lac	*Trametes pubescens*	0.001	pH 6.9, 25 °C, 24 h.	100	>90	[57]
17-α-ethynyl estradiol	Lac	*Trametes pubescens*	0.001	pH 6.9, 25 °C, 24 h.	100	>90	[57]
Sulfamethoxazole	Lac	*Phanerochaete chrysosporium*	10	pH 4.5, 30 °C, 48 h.	6076	50	[58]
	Lac	*Pycnoporus sanguineus*	10	30 °C, 72 h.	170	29	[59]
17-β-estradiol	Lac	*Trametes hirsuta*	5	pH 5, 25 °C, 120 min.	5000	99	[50]
	Lac	*Trametes pubescens*	0.001	pH 6.9, 25 °C, 24 h.	100	>90	[57]
Tetracycline ^1^ Oxytetracycline ^2^	MnP	*Phanerochaete chrysosporium*	50	pH 4.8, 0.1 mM Mn^2+^, 0.1 mM H_2_O_2_, 37 °C, 4 h.	40	73 ^1^ 84 ^2^	[60]
Acetaminophen	Lac	*Bjerkandera adusta TBB-03*	20	pH 5–7, 25 °C, 2 h.	270	100	[61]
	HRP	*Horseradish*	6	pH 7.4, 400 μM H_2_O_2_, 25 °C, 4 h.	12800	100	[8]
Triclosan	Lac	*Trametes versicolor*	3	pH 6, 25 °C, 4 h.	2000	52	[62]
Doxorubicin	Lac	*Trametes Versicolor*	0.25	pH 7, 30 °C, 2 h.	900	100	[21]
Imipramine	Lac	*Paraconiothyrium variabile*	0.12	pH 5, 37 °C, 6 h.	1600	98	[20]

**Table 2:** Characteristics of redox mediators used in treatment of micropollutants by Lac-mediator systems.

Oxidation Mechanism	Redox Mediator	Origin	Type of Mediator	Free Radical Generated
Electron transfer	2,2′-azino-bis (3-ethylbenzothiazoline-6-sulfonic acid) diammonium salt	Synthetic	ABTS	ABTS^•+^ ABTS^2+^
Hydrogen atom transfer	Hydroxybenzotriazole	Synthetic	N–OH	=N–O^•^ Aminoxyl
	N-hydroxyphthalimide	Synthetic	N–OH	=N–O^•^ Aminoxyl
	Violuric acid	Natural	N–OH	=N–O^•^ Aminoxyl
	Vanillin	Natural	C_6_H_4_(OH)(OCH_3_)	C_6_H_5_O^•^ Phenoxyl
	Syringaldehyde	Natural	C_6_H_4_(OH)(OCH_3_)	C_6_H_5_O^•^ Phenoxyl
	Acetosyringone	Natural	C_6_H_4_(OH)(OCH_3_)	C_6_H_5_O^•^ Phenoxyl
	*p*-coumaric acid	Natural	C_6_H_4_(OH)(OCH_3_)	C_6_H_5_O^•^ Phenoxyl
Ionic oxidation	2,2,6,6-tetramethylpiperidinyloxyl	Synthetic	N-O	N=O^•^ Oxo-ammonium

**Table 3:** Examples of biodegradation of pharmaceutical micropollutants by Lac coupled with reaction mediators.

Compound	Lac Source	Mediator	Reaction Conditions	Enzyme Load (U/L)	Efficiency (%)	Ref.
Diclofenac	*Trametes versicolor*	1 mM of HBT ^1^ and SA ^2^	0.1 mg/L PhAC. 25 °C, 24 h.	1440	>95 ^1^ 80 ^2^	[52]
Carbamazepine	*Trametes versicolor*	0.018 mM of ABTS	1 mg/L PhAC. pH 6, 35 °C, 24 h.	60	95	[56]
Sulfamethoxazole	*Trametes versicolor*	0.5 mM ABTS, SA and AS	20-25 mg/L PhAC. pH 6–7, 25 °C, 2–6 h.	560	100	[81]
Tetracycline Oxytetracycline	*Pycnoporus* sp. *SYBC-L10*	1 mM of ABTS	50 mg/L PhAC. pH 6, 5 min, 0 °C.	10,000	100	[41]
Chloramphenicol	*Trametes hirsuta*	0.5 mM of SA, vanillin, ABTS and α-naphthol	10 mg/L PhAC. pH 5, 25 °C, 48 h.	50,220	100	[51]
Ketoconazole	*Trametes versicolor*	1 mM of HBT	300 mg/L PhAC. pH 4.5, 45 °C, 6 h.	1000	98	[83]
Naproxen	*Pleurotus ostreatus*	1 mM of ABTS	0.5 mg/L PhAC. 25 °C, 8 h.	0.26	80	[82]
Olsalazine	*Aspergillus aculeatus*	2 mM of ABTS ^1^, *p*-Coumaric acid ^2^ and HBT ^3^	100 mg/L PhAC. pH 5, 29 °C, 48 h.	-	99 ^1^ 98 ^2^ 94 ^3^	[84]
Atenolol	*Trametes versicolor*	0.5 mM of TEMPO	2.7 mg/L PhAC. pH 7, 25 °C, 4 h.	5000	80	[85]

**Table 4:** Examples of the removal of pharmaceutical compounds by immobilized enzyme.

Support Material	Enzyme	Immobilization Method	PhAC	Reaction Conditions	Efficiency (%)	Ref.
Poly(l-lactic acid)-co-poly(ε-caprolactone) nanofibers	Lac from *Trametes versicolor*	Encapsulation	Naproxen	1 mg/L PhAC. pH 5, 25 °C, 24 h, 100 rpm.	90	[93]
Poly(l-lactic acid)-co-poly(ε-caprolactone) nanofibers	Lac from *Trametes versicolor*	Encapsulation	Diclofenac	1 mg/L PhAC. pH 3, 25 °C, 24 h, 100 rpm.	90	[93]
Polyvinylidene fluoride membrane with multi-walled carbon nanotubes	Lac from *Trametes hirsuta*	Covalent bonding		5 mg/L PhAC. pH 5, 25 °C, 4 h.	95	[94]
Titania nanoparticles	Lac from *Pycnoporus sanguineus CS43*	Covalent bonding		10 mg/L PhAC. pH 4, 25 °C, 4 h.	50	[101]
Micro-biochar from pine wood (PW) and pig manure (PM)	Lac from *Trametes versicolor*	Covalent bonding		0.5 mg/L PhAC. pH 6.5, 25 °C, 5 h (PW) or 2 h (PM).	99	[98]
Chitosan macro-beads	Lac from *Trametes versicolor*	Covalent bonding		50 mg/L PhAC. pH 3, 25 °C, 4 h, 1:1 M ratio for ABTS:drug.	90	[49]
CLEA	Lac from *Trametes versicolor*	Cross-linking		0.001 mg/L PhAC. pH 5, 24 h, 22 °C.	90	[99]
Polyacrylonitrile−biochar composite nanofibrous membrane	Lac from *Trametes versicolor*	Covalent bonding		0.2 mg/L PhAC, pH 4, 8 h, 35 °C.	73	[102]
Polyacrylonitrile−biochar composite nanofibrous membrane	Lac from *Trametes versicolor*	Covalent bonding	Chlortetracycline	0.2 mg/L PhAC, pH 4, 8 h, 35 °C.	63	[102]
Polyvinylidene fluoride membrane with multi-walled carbon nanotubes	Lac from *Trametes hirsuta*	Covalent bonding	Carbamazepine	5 mg/L PhAC. pH 5, 25 °C, 48 h.	27	[94]
Magnetite nanoparticles	HRP LiP	Adsorption		0.35 mg/L PhAC. pH 3, 55 °C, 3 days.	100	[103]
Polyimide aerogels	Lac from *Trametes versicolor*	Covalent bonding		0.02 mg/L PhAC. pH 3, 25 °C, 24 h, 200 rpm.	74	[104]
Pinewood nanobiochar	Lac from *Trametes versicolor*	Adsorption		0.02 mg/L PhAC. pH 3.5, 25 °C, 24 h, 200 rpm.	80	[92]
Polyamide/polyethylenimine nanofibers	Lac from *Trametes versicolor*	Covalent bonding	Triclosan	10 mg/L PhAC. pH 7, 25 °C, 20 h, 80 rpm.	74	[105]
Titania nanoparticles	Lac from *Pycnoporus sanguineus CS43*	Covalent bonding	Acetaminophen	10 mg/L PhAC. pH 4, 25 °C, 4 h.	90	[101]
Commercial silica gel particles	Lac from *Trametes versicolor*	Covalent bonding	Sulfamethoxazole	20 mg/L PhAC. pH 7, 25 °C, 0.5 h. 520 μM of ABTS.	53	[106]
Commercial silica gel particles	Lac from *Trametes versicolor*	Covalent bonding	Amoxicillin	20 mg/L PhAC. pH 7, 25 °C, 4 h. 520 μM of ABTS.	80	[106]
M-CLEA	Lac from *Cerrena unicolor*	Cross-linking	Tetracycline	100 mg/L PhAC. pH 6, 25 °C, 48 h.	100	[89]
Mesostructured cellular foam	Lac from *Trametes versicolor*	Adsorption		1 mg/L PhAC. pH 5, 25 °C, 1 h.	100	[107]
Bentonite-derived mesoporous materials	Lac from *Trametes versicolor*	Adsorption		10 mg/L PhAC. 30 °C, 3 h.	60	[100]
Cellulose beads	Lac from *Trametes versicolor*	Covalent bonding	Indole	15 mg/L PhAC. pH 5, 30 °C, 18 h.	100	[108]
Polypropylene beads	Lac from *Myceliophthora thermophila*	Adsorption	Morphine	1 mg/L PhAC. pH 6, 25 °C, 0.5 h.	100	[109]
Graphene oxide and alginate matrix	Lac from *Aspergillus niger*	Adsorption/entrapment	Cetirizine dihydrochloride	20 mg/L PhAC. pH 4.5, 25 °C, 1 h.	100	[23]
Pristine few layers graphene	Lac from *Trametes versicolor*	Adsorption	Labetalol hydrochloride	1 mg/L PhAC pH 7, 25 °C, 1.5 h. 5 μM of ABTS.	100	[110]

## Data Availability

Not applicable.

## Abbreviations

ABTS	2,2′-azino-bis (3-ethylbenzothiazoline-6-sulfonic acid) diammonium salt
HWW	Hospital wastewater
APAP	Acetaminophen
IBP	Ibuprofen
APTES	3-aminopropyltriethoxysilane
Lac	Laccase
AS	Acetosyringone
LiP	Lignin peroxidase
ATL	Atenolol
M-CLEA	Magnetic cross-linked enzyme aggregates
CAP	Chloramphenicol
MCF	Mesostructured cellular foam
CBZ	Carbamazepine
MF	Microfiltration membrane
CLEA	Cross-linked enzyme aggregate
MnP	Manganese peroxidase
DCF	Diclofenac sodium
MWCNT	Multi-walled carbon nanotubes
DDM	Dichlorophen
NF	Nanofiltration
DHQ	5,7-diiodo-8-hydroxyquinoline
NPX	Naproxen
DMBQ	2,6-dimethoxy-1,4-benzoquinone
NSAID	Non-steroidal anti-inflammatory drugs
DOX	Doxorubicin
OTC	Oxytetracycline
EC	Emerging contaminants EC
PhAC	Pharmaceutical active compounds
EDG	Electron-donating groups
PLCL	Poly(l-lactic acid)-co-poly(ε-caprolactone)
EMR	Enzymatic membrane reactor
PVDF	Polyvinylidene fluoride
ET	Electron transfer
SA	Syringaldehyde
EWG	Electron withdrawing groups
SMX	Sulfamethoxazole
FLG	Few-layer graphene
TC	Tetracycline
GAC	Granular activated carbon
TEMPO	2, 2, 6, 6-tetramethyl-1-piperidinyloxy
GO	Graphene oxide (GO)
TNC	Trinuclear copper cluster
HA	Humic acids
UF	Ultrafiltration
HAT	Hydrogen atom transfer
VLA	Violuric acid
HBR	Hybrid bioreactor
VP	Versatile peroxidase
HBT	Hydroxybenzotriazole
WRF	White-rot fungi
HPI	N-hydroxyphthalimide
WWTP	Conventional wastewater treatment plants
HRP	Horseradish peroxidase
